# Identification of Soil Types and Salinity Using MODIS Terra Data and Machine Learning Techniques in Multiple Regions of Pakistan

**DOI:** 10.3390/s23198121

**Published:** 2023-09-27

**Authors:** Yasin Ul Haq, Muhammad Shahbaz, Shahzad Asif, Khmaies Ouahada, Habib Hamam

**Affiliations:** 1 Department of Computer Science, University of Engineering and Technology, Lahore 39161, Pakistan; 2 Department of Computer Engineering, University of Engineering and Technology, Lahore 39161, Pakistan; m.shahbaz@uet.edu.pk; 3 Department of Computer Science, New Campus, University of Engineering and Technology, Lahore 39161, Pakistan; shehzad@uet.edu.pk; 4 Department of Electrical and Electronic Engineering Science, School of Electrical Engineering, University of Johannesburg, Johannesburg 2006, South Africa; kouahada@uj.ac.za; 5 College of Computer Science and Engineering, University of Hail, Hail 55476, Saudi Arabia; habib.hamam@umoncton.ca

**Keywords:** remote sensing, soil types, soil salinity, spectral signature, random forest, MODIS Terra data, gradient boosting

## Abstract

Soil, a significant natural resource, plays a crucial role in supporting various ecosystems and serves as the foundation of Pakistan’s economy due to its primary use in agriculture. Hence, timely monitoring of soil type and salinity is essential. However, traditional methods for identifying soil types and detecting salinity are time-consuming, requiring expert intervention and extensive laboratory experiments. The objective of this study is to propose a model that leverages MODIS Terra data to identify soil types and detect soil salinity. To achieve this, 195 soil samples were collected from Lahore, Kot Addu, and Kohat, dating from October 2022 to November 2022. Simultaneously, spectral data of the same regions were obtained to spatially map soil types and salinity of bare land. The spectral reflectance of band values, salinity indices, and vegetation indices were utilized to classify the soil types and predict soil salinity. To perform the classification and regression tasks, the study employed three popular techniques in the research community: Random Forest (RF), Ada Boost (AB), and Gradient Boosting (GB), along with Decision Tree (DT), K-Nearest Neighbor (KNN), and Extra Tree (ET). A 70–30 test train validation split was used for the implementation of these techniques. The efficacy of the multi-class classification models for soil types was evaluated using accuracy, precision, recall, and f1-score. On the other hand, the regression models’ performances were evaluated and compared using R-squared (R${}^{2}$), Mean Squared Error (MSE), Mean Absolute Error (MAE), and Root Mean Squared Error (RMSE). The results demonstrated that Random Forest outperformed other methods for both predicting soil types (accuracy = 65.38, precision = 0.60, recall = 0.57, and f1-score = 0.57) and predicting salinity (R${}^{2}$ = 0.90, MAE = 0.56, MSE = 0.98, RMSE = 0.97). Finally, the study designed a web portal to enable real-time prediction of soil types and salinity using these models. This web portal can be utilized by farmers and decision-makers to make informed decisions regarding soil, crop cultivation, and agricultural planning.

## 1. Introduction

Soil constitutes a blend of minerals, organic substances, living organisms, fluids, and gases, collectively fostering life, and playing a pivotal role in the Earth’s ecosystem. The composition of soil can experience daily fluctuations due to a numerous factors, including water availability, agricultural practices, soil classification, and various other variables [1]. The development of innovative farming methods for effective management to stop soil degradation depends critically on precise knowledge of the spatial variability of agricultural soil attributes [2,3,4]. In addition to other characteristics, soil type regulates the mobility, retention, and concentration of dissolved compounds in soil. As a result, it affects crop yield and nutrient balance in the rhizosphere [5,6]. In environmental and agricultural domains, the soil type plays a pivotal role as a fundamental input for modeling and evaluation purposes [7,8]. Existing soil-type maps usually have a low level of detail and a coarse resolution, which makes it difficult to model and manage resources effectively in croplands [4,9]. Measurements of soil types taken on the ground are labor and money-intensive. As a result, numerous scientists have worked extremely hard to create reliable and economical methods that deliver updated and better soil-type maps [10,11]. Through the integration of Remote Sensing (RS) data and Digital Soil Mapping (DSM), scientists have effectively demonstrated the promise of a resilient, efficient method for mapping a wide range of soil characteristics. RS technology effectively addresses the limitations encountered in conventional soil mapping, leading to substantial reductions in labor requirements both in the field and the laboratory [11,12,13,14,15,16,17,18]. The application of multispectral satellite data from space, particularly in the near-infrared and optical bands (VNIR-SWIR), offers possibilities for the quantification of various soil properties, yielding outcomes that exhibit varying degrees of success success [19,20,21].

Soil salinity, resulting from both human-induced actions and natural sources, presents a substantial environmental hazard, especially in arid and semi-arid regions worldwide. The proliferation of salt in soil poses a serious threat to global agriculture and crop yields, underscoring the urgent need to swiftly and accurately identify areas affected by salinity. This is crucial for sustaining soil health and ensuring a stable food supply [22]. Traditional methods for measuring soil salinity encompass laboratory analysis and on-site field surveys. Since the 1960s, the utilization of both black-and-white and color aerial imagery has been employed for the identification of soil salinity and the collection of data related to diverse surface features on Earth [23]. RS is a technique that relies on the capture of electromagnetic energy from sunlight, which is reflected off the Earth’s surface, to collect data about various features on our planet, offering different levels of detail depending on the application and technology employed [23]. Remote sensing devices can be categorized as either passive or active. Passive devices utilize natural Electromagnetic Radiation (EMR) sources, whereas active devices require additional EMR for remote detection. When applied to multisource datasets, Machine Learning (ML) models prove to be more effective than traditional statistical models [24,25,26,27]. ML models are employed to quantify the relative importance of covariates from different sources that govern soil variability [28]. Several studies [24,25,29] have shown that when ML models are applied to the same data source, their prediction outcomes can exhibit substantial differences. As a result, it becomes essential to assess the performance of different models when dealing with multisource datasets, especially in domains characterized by significant intrinsic and extrinsic variability [25]. Soil type and crop yield estimation may be possible with better accuracy at higher geographic resolutions using a method that simultaneously combines multisensor RS data along with environmental variables [30,31,32].

In a study conducted in Yanqi Basin, Xinjiang, China, researchers [33] utilized both ANN and SVM regression algorithms to assess soil salinity and its spatial distribution based on four parameters: Groundwater Depth, soil backscattering coefficient ($\sigma_{soil}^{0}$), Surface Evapotranspiration, and Salinity Index. The results of statistical evaluations during both the training and testing phases revealed that the SVM technique, employing a nonlinear transfer function, outperformed the ANN technique. In the training dataset, SVM achieved an R${}^{2}$ value of 0.82 with an RMSE of 2.01, while in the testing dataset, it obtained an R${}^{2}$ value of 0.88 with an RMSE of 1.36. Conversely, the ANN technique exhibited slightly lower performance, with an R${}^{2}$ value of 0.79 and an RMSE of 2.20 in the training dataset, and an R${}^{2}$ value of 0.68 with an RMSE of 2.25 in the testing dataset.

Similarly de Oliveira Morais et al. [34], conducted research in Brazil to analyze soil texture by employing digitally scanned images. They utilized 177 topsoil samples to annotate these scanned images and subsequently utilized Least-Squares Support Vector Machine Regression (LSSVMR) for topsoil texture analysis. Notably, the LSSVMR method outperformed others, achieving correlations exceeding 90% in estimating topsoil texture.

In a similar attempt, Khallouf et al. [35] worked to predict the topsoil texture particle distribution and made digital soil maps for the Al-Ghab plain, Syria. Three methods of MLR, backward elimination, forward selection, and stepwise selection were used for the selection of predictors. The backward elimination method was declared the best method for predictor selection for all three soil textures as it gave the highest R${}^{2}$ values for clay, silt, and sand (45.5, 28.6, 31.3%). They also concluded that the Multi-Resolution Valley Bottom Flatness Index (MRVBF), percentage Topographic Wetness Index (TWI), Modified Soil Adjusted Vegetation Index (MSAVI), and Green Soil Adjusted Vegetation Index (GSAVI) were the best predictors for predicting the soil texture. Similarly, Wang et al. [36] employed remote sensing data and machine learning techniques to estimate soil salt content. They conducted their analysis with a dataset of 48 samples, focusing on soil samples collected from a depth of 0–20 cm. They used Normalized Difference Index (NDI), Difference Index (DI), Salt Index (SI), and Ratio Index (RI) parameters to build the PSO-SVM model. The results showed that they mapped the soil salinity with R${}^{2}$ = 0.66.

According to Swain et al. [37], PLSR, SVR, and RF models were utilized to predict the silt, clay, and sand contents based on both S2 spectra and laboratory measurements. The results described that the ensemble modeling approach estimated the soil content with high R${}^{2}$ values of sand, silt, and clay (0.62, 0.54, 0.54). In a similar attempt, Wang et al. [38] used 35 indices, and four machine learning models (CNN, PLSR, RF, and SVM) were implemented to predict the salinity of soil in the Aksu district, Southern Xinjiang Province, northwestern China. The RFR model performed best with R${}^{2}$ = 0.75 and RMSE = 7.33%. Wang et al. [39] predicted the soil salinity in the Tarim River Basin of southern Xinjiang, China at five depth levels with the help of an RF model. The accuracy of the RF model was higher at the surface (R${}^{2}$ = 0.65) than deeper (R${}^{2}$ = 0.63) or the transitional zone (R${}^{2}$ = 0.55). Similarly, Aksoy et al. [22] developed a model for mapping the soil salinization using Sentinel-2A and Landsat-8 OLI satellite data with three different ML algorithms RF, CART, and SVR. The results of the CART model for Sentinel-2A data and Landsat 8 data (R${}^{2}$ = 0.98, R${}^{2}$ = 0.96) were slightly better than the RF model (R${}^{2}$ = 0.96, R${}^{2}$ = 0.94) while mapping the soil salinity. In a similar attempt, Cheng et al. [40] utilized the Landsat data to estimate the salinity of soil using Multiple Linear Regression (MLR) and Partial Least Square Regression (PLSR) models in different depths across the Yellow River Delta, China. The results proved that PLSR performed (R${}^{2}$ = 0.62) better than MLR (R${}^{2}$ = 0.45).

Ijaz et al. [23] used Landsat 8 imagery and calculated the salinity and vegetation indices for characterizing and evaluating the salinity of soil in Kot Addu, Pakistan. According to remote sensing data, in comparison to normal soils, saline soils have greater reflectance in the visible and near-infrared spectra. Results showed that the vegetation indices were more useful in mapping soil salinity than salinity indices in places where there is vegetation. The effectiveness of indices changed with the salt levels, as well as the type and amount of vegetation; therefore, it is advised to utilize many indices as a single index might not always produce the best results. They mapped the soil salinity with R${}^{2}$ = 0.54 and also generated the salinity maps. Recently, Haq et al. [41] proposed a model for determining the soil types using remote sensing data. Landsat 8 images were taken in order to classify soil types using three machine learning models RS, SVM, and Logistic Model Tree (LMT). RF produced the best results, with an accuracy rate of 86.61%. According to their findings, NDVI and SAVI’s spectral characteristics were more useful for determining the type of soil.

Hence, the primary objective of the present research is to identify soil types and predict soil salinity based on spectral reflectance, vegetation indices, and salinity indices obtained from bare land using Modis Terra data. This study relies on satellite data collected over Lahore, Kot Addu, and Kohat in Pakistan. The main emphasis is to evaluate the responsiveness of spectral band reflectance, vegetation indices, and salinity indices to classify the soil types and determine the salinity level. The objectives of this research encompass:

1. To determine which ML algorithm is more effective for soil type classification and salinity detection from RF, DT, GB, AB, and ET.
2. To map the soil types and salinity using the reflectance of band values, calculated vegetation indices, and salinity indices from MODIS Terra data.
3. To determine which crop is more suitable based on certain soil types and salinity values.
4. To identify the soil type and salinity by just entering the geo-coordinates of the location.

This paper is organized into four primary sections. In Section 2, the materials and methodology of this research are given. Section 3 contains results. Section 4 presents a detailed discussion of the results obtained in the study. Finally, Section 5 contains our conclusions.

## 2. Materials and Methods

### 2.1. Study Area

**Figure 1:**
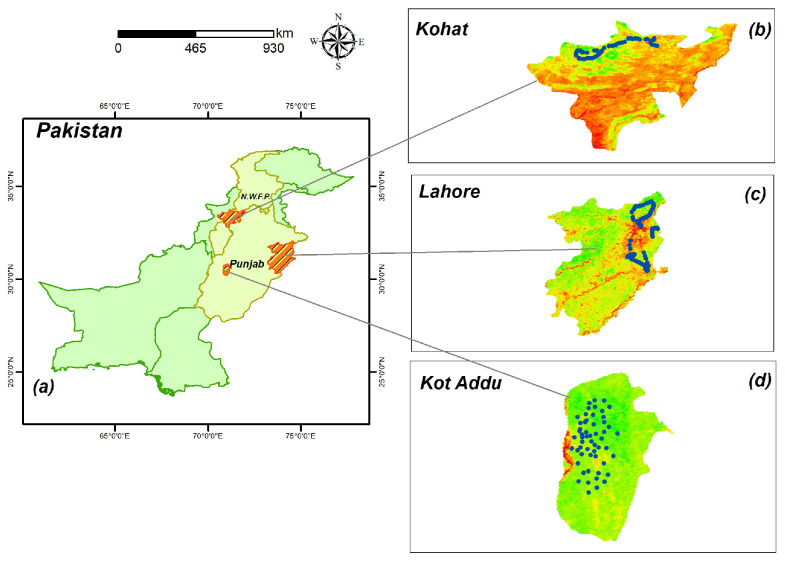
The geographical positioning of the three study regions Kohat, Lahore, and Kot Addu.

Three semi-arid regions in Pakistan, namely Lahore, Kot Addu, and Kohat, were selected as the study sites. Figure 1 illustrates the geographical representation of the study area. Lahore, covering a total area of 1772 km${}^{2}$, holds the dual distinction of being the provincial capital and the second-largest city in Pakistan. The weather in Lahore is semi-arid. Winter temperatures average 13.09 °C to 24.71 °C, while summer temperatures range from 25.73 °C to 36.09 °C. The average annual precipitation ranges from 17.04 mm in the winter to 88.28 mm in the summer. The River Ravi, which originates in the Himalayan region, is the main river that flows through the study area. Lahore City is located at an average elevation of 208–213 m and has a flat terrain. The crops grown in the district include wheat, bajra, rice, sugarcane, masoor, moong, maash, sunflower, rapeseed, mustard, maize, jowar, and tobacco [42].

The area of Kot Addu is situated nearly exactly in the geographic center of Pakistan in the Muzaffargarh District of Punjab Province [23]. In the alluvial plain that encircles the city, the main crops grown include sugarcane, cotton, and wheat; smaller-scale crops include moong, rice, masoor, ground nuts, bajra, maize, mash, jawar, and oil seeds (rapeseed and sunflower). Dates, mangoes, pomegranates, and citrus are the principal fruit trees cultivated. In many citrus and mango fields, pears, phalsa, bananas, jaman, and dates occupy only a small fraction of the cultivated area [23]. The majority of the agricultural land is mild to moderately salinated. Aridity and warm winters and summers characterize Kot Addu’s climate. The city has seen some of Pakistan’s most severe weather [23]. The peak temperature ever recorded in this region was approximately 51 °C (123.8 °F), while the lowest temperature ever recorded was around 1 °C (33.8 °F). On average, the annual precipitation in this area amounts to about 127 mm (5.0 inches) of rainfall [23].

Kohat Division consists of five districts: Hangu, Karak, Kohat, Kurram, and Orakzai. Kohat Division has a total area of 12,377 km${}^{2}$ (4779 sq mi). The region of Kohat is characterized by its hilly terrain, with an average elevation of 1500 m (4900 feet) above sea level. It experiences hot weather from May to September, with June being the hottest month, with temperatures around 40 °C (maximum) and 27 °C (minimum). From October to February, the weather becomes pleasant but cold and severe in winter, often accompanied by the “Hangu Breeze”—wind blowing down the Miranzai valley towards Kohat for extended periods. The average annual rainfall is approximately 638 mm. Major crops cultivated in the area include onion, wheat, garlic, barley (Rabi crops), groundnut, maize, chari, rice, sugarcane, and bajra [43].

### 2.2. Satellite Data

For this study, MODIS Terra images, i.e, MODO9A1/v061 (MODIS/Terra Surface Reflectance 8-Day L3 Global 500 m SIN Grid) were downloaded from USGS Earth Explorer, https://earthexplorer.usgs.gov/ (accessed on 10 December 2022), which is a free and open-source platform for downloading satellite images for remote sensing. Images were acquired between October 2022 to November 2022. Five images were downloaded with cloud cover less than 3%.

### 2.3. Soil Samples

During the period between 1 October 2022, and November 2022, a comprehensive collection of soil samples was carried out, ensuring their random distribution across the study area. These samples were taken from the upper 0–15 cm layer of the soil surface, using an auger, and the precise coordinates of each sampling location were recorded using Google Maps. Subsequently, the collected soil samples were carefully placed in polybags and sent to the Agricultural Research Institute Tarnab, Peshawar for further in-depth analysis.

### 2.4. Spectral Indices

Using ArcGIS 10.8, we calculated the reflectance of band values, i.e, Bands 1–7, vegetation indices and salinity indices based on literature review. The mathematical expression for these vegetation and salinity indices are displayed in Table 1.

**Figure 2:**
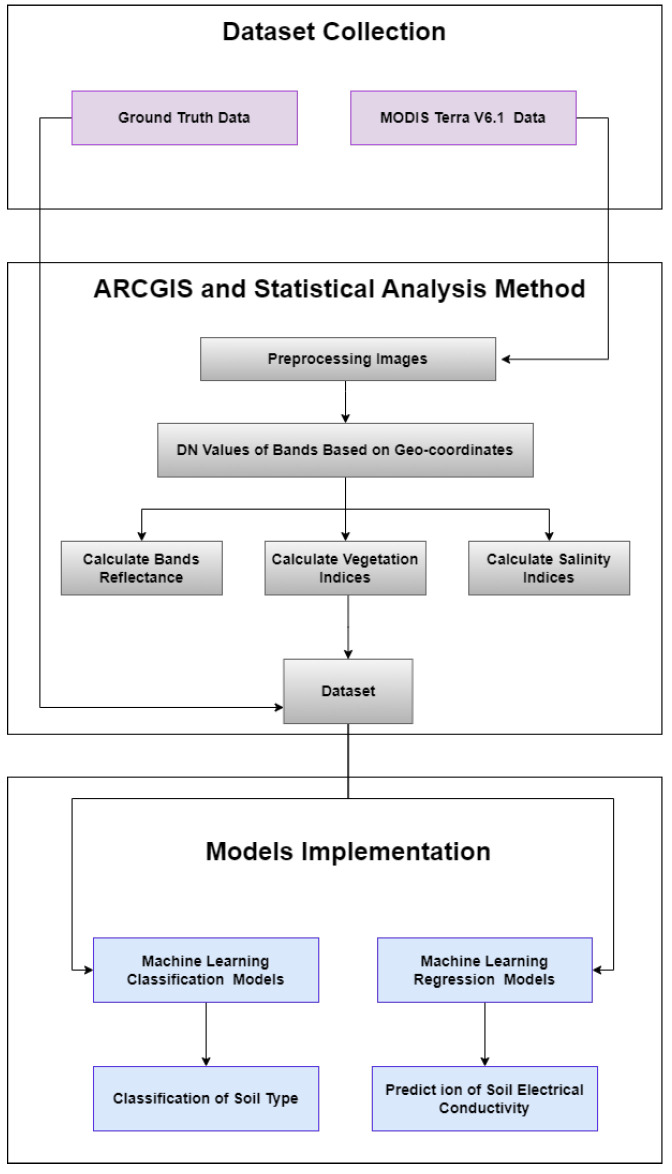
Methodology of the current study.

After calculating the indices, the combined dataset was made from reflectance of bands (1–7), vegetation indices, spectral indices, and laboratory results. Several preprocessing techniques [41] were implemented before the training of ML models. To classify soil types, the ML models utilized input features consisting of reflectance values from seven bands and four vegetation indices, with the target class labeled as “soil type”. In contrast, for estimating soil salinity levels, the ML models were trained using reflectance values from seven bands in combination with eight salinity indices. The methodology used to classify the soil types and detection of soil salinity using MODIS Terra data is shown in Figure 2. The implementation was carried out in Python using the sklearn library within a Jupyter Notebook.

### 2.5. Machine Learning Models

Various ML models have already been implemented to assess soil types and salinity, with AB, GB, and RF being among the commonly utilized techniques. In the current study, we have preferred six ML models (RF, GB, AB, DT, KNN, and ET) to map the soil types and soil salinity, more details of these models are given in the following subsection.

#### 2.5.1. Random Forest Algorithm

Initially introduced by Breiman [54] and Cutler and Stevens [55], RF is an ensemble ML technique widely applied in various applications [54]. In this method, multiple decision tree classifiers are simultaneously fitted on different sub-samples of the dataset. The final outcome is determined by a majority vote or averages. The RF algorithm effectively addresses the problem of overfitting while enhancing prediction accuracy [56]. Consequently, an RF learning model with multiple decision trees typically outperforms a model based on a single decision tree [57], achieving this by combining “bootstrap aggregation (bagging)” [58] and “random feature selection” [59]. This combination results in a collection of decision trees with controlled variance. RF exhibits strong performance for both categorical and continuous variables, rendering it a versatile solution for both classification and regression tasks [60]. To achieve optimal results with the RF model, several parameters were carefully selected. The number of decision trees (ntree) was set to 26, indicating that the model comprises 26 individual decision trees. The max_depth parameter was set to 14, governing the maximum depth or complexity of each decision tree within the forest. Finally, the random_state parameter was established at 8, ensuring result reproducibility by providing a fixed seed for the random number generator.

#### 2.5.2. Gradient Boosting Algorithm

Similarly, like Random Forests [54] discussed earlier, GB is another ensemble learning technique that builds a final model by combining multiple individual models, often based on decision trees. Comparable to how neural networks utilize gradient descent to optimize weights [61], GB utilizes the gradient to minimize the loss function. A specific variant of gradient boosting, known as Extreme Gradient Boosting (XGBoost), takes a more meticulous approach to approximate the optimal model [56]. It accomplishes this by computing enhanced regularizations (L1 and L2) to reduce overfitting, enhance model generalization, and consider second-order gradients of the loss function to minimize errors. Gradient Boosting finds applications in both classification and regression tasks [56]. To achieve the best results with the GB model, several parameters were carefully selected. The number of decision trees (ntree) was set to 8, indicating that the model comprises 8 individual decision trees. The max_depth parameter was configured to 7, governing the maximum depth or complexity of each decision tree within the ensemble. Finally, the random_state parameter was established at 10, ensuring result reproducibility by providing a fixed seed for the random number generator.

#### 2.5.3. Extra Trees Algorithm

When dealing with supervised classification and regression problems, ET stands out as a tree-based ensemble technique [62,63]. In contrast to certain alternative tree-based clustering methods, ET adopts the conventional top-down approach for constructing a sequence of unprocessed gradient or regression trees. Its distinguishes itself by randomly selecting cut-points to partition nodes and utilizing the complete learning dataset for tree construction [54]. The ultimate prediction is established by consolidating the predictions of individual trees, employing either arithmetic averaging for regression challenges or majority voting for classification tasks [64]. According to John et al. [65], ET employs a strategy of utilizing random subsets of features to train each base estimator. To achieve optimal results with the ET model, specific parameters were fine-tuned. The number of decision trees (ntree) was adjusted to 39, indicating that the model encompasses 39 individual decision trees. Furthermore, the random_state parameter was configured to 42, ensuring the reproducibility of results by using a fixed seed for the random number generator.

#### 2.5.4. Ada Boost Algorithm

AdaBoost, an ensemble learning method, employs an iterative procedure to enhance the performance of weak classifiers by learning from their mistakes. Introduced by Yoav Freund et al. [66], it is also known as “metalearning”. Unlike random forests, which use “parallel ensembling”, AdaBoost utilizes “sequential ensembling”. By combining multiple weak classifiers, it creates a powerful classifier with high accuracy. Referred to as an adaptive classifier, AdaBoost significantly improves classifier efficiency, but it may occasionally lead to overfitting. For binary classification tasks, it works exceptionally well when used to boost the performance of decision trees [56]. However, it is worth noting that AdaBoost is susceptible to noisy data and outliers. To achieve better results, certain parameters were tuned in the model, specifically for the number of decision trees (ntree), random_state, max_depth, and base_estimator. The ntree parameter was set to 26, indicating that the model consists of 26 individual decision trees. The random_state parameter was set to 4, ensuring reproducibility of the results by providing a fixed seed for the random number generator. Additionally, a decision tree with a max_depth of 14 and a random_state of 40 was set as the base_estimator. This decision tree serves as the individual classifier within the ensemble model.

#### 2.5.5. K-Nearest Neighbors Algorithm

K-Nearest Neighbors (KNN) [67] is a method for “lazy learning” or “instance-based learning”, also known as non-generalizing learning. Unlike approaches that focus on constructing comprehensive internal models, KNN retains all instances that belong to the training set in an n-dimensional space. KNN operates by analyzing existing data and classifying new data points based on similarity metrics, such as the Euclidean distance function [56]. In the KNN approach, each point seeks input from its k-nearest neighbors, who cast their votes, and the classification is determined by a simple majority. The accuracy of KNN dependent on the quality of the data and exhibits a degree of resilience to noisy training data. The primary challenge with KNN lies in selecting the optimal number of neighbors to consider, which, in our case, is K = 1. KNN finds applications in both classification and regression tasks.

#### 2.5.6. Support Vector Algorithm

SVMs, which were first introduced by Cortes and Vapnik [68], are learning models often used for distribution estimation, regression, and classification problems. Using nonlinear methods, SVMs turn the original independent variables into a higher or infinite dimensional feature space. They aim to achieve a better separation [69]. It is commonly used in many remote sensing applications. In the case of multi-class classification, as data are not linearly separable, SVM used kernel functions, i.e., linear, polynomial, radial basis, and sigmoid, each requires a unique set of parameters, and the appropriate choice and parameterization of these kernels affects the SVM’s accuracy [68]. To avoid overfitting and to control the trade-off between margins and training mistakes, tuning parameters C, also known as the penalty factor, were used [70]. Using SVM, any problem can be solved by choosing a good kernel function [41]. In this research, The Radial Basis Function (RBF) kernel function was picked for both classification and regression problems. For the RBF kernel, two important parameters need to be specified: C and gamma. The C parameter controls the trade-off between maximizing the margin and minimizing the classification/regression errors. A larger C value allows for a narrower margin and can lead to potentially overfitting the data. In the current study, the value of C was set to 22, indicating a relatively higher emphasis on maximizing the margin and potentially reducing the training error. However, it is important to note that the optimal value of C may vary depending on the dataset and the specific problem. The gamma parameter controls the shape of the RBF kernel function and determines how far the influence of a single training example reaches. A higher gamma value causes the RBF kernel to have a sharper peak, resulting in a more localized influence of each training example on the decision boundary, and in our case, the value of gamma was 5.

#### 2.5.7. Decision Tree Algorithm

A popular non-parametric supervised learning approach is the DT [71]. Both the classification and regression tasks are carried out using DT learning techniques [56]. DT algorithms that are popular include ID3 [72], C4.5 [71], and CART [73]. Tuning the parameters of a decision tree algorithm, such as setting the maximum depth and random state, can help improve its performance and achieve better results. By tuning these parameters, we specified a maximum depth of 15 for the decision tree, allowing it to capture more complex patterns in the data without overfitting. Additionally, setting the random state to 29 ensures that the random aspects of the algorithm are consistent across different runs [56].

### 2.6. Evaluation Metrics

Band values and vegetation indices were provided as input to these classifiers, and the target value observed was soil types. A total of one hundred ninety-five tuples data was divided into testing and training sets at a ratio of 70% and 30%. We calculated the *accuracy*, *recall*, *precision*, and *f1*-score for classification of soil type. *Accuracy* explains how the model perform across all classes [74], the formula of *accuracy* is given in Equation (1).

(1) $$Accuracy=\frac{TP+TN}{TP+TN+FP+FN}$$

Precision is a metric that assesses the accuracy of positive predictions. It quantifies the ratio of correctly classified positive samples to the total number of samples classified as positive, whether they were classified correctly or incorrectly [74]. The formula of precision is given in Equation (2).

(2) $$Precision=\frac{TP}{TP+FP}$$

Recall evaluates a model’s capability to accurately identify all relevant instances of a specific class. It represents the proportion of positively classified instances that are correctly identified out of the total number of positive instances [74] as given in Equation (3).

(3) $$Recall=\frac{TP}{TP+FN}$$

The f1 score is derived from the harmonic mean of precision and recall. By combining both precision and recall, the f1 score offers a single value that represents the balance between these two metrics. Its range lies between 0 and 1, where a score of 1 signifies perfect precision and recall, while a score of 0 indicates poor performance [75]. The mathematical expression of the f1 score is given in Equation (4).

(4) $$f1-score=\frac{2\times Precision\times Recall}{Precision+Recall}$$

To predict the soil salinity, we used band values and salinity indices as input parameters. Soil samples were collected 1 October 2022 to November 2022 and the whole dataset was split into training and testing sets with the ratio of 70% to 30%. R${}^{2}$, MAE, RMSE, and MSE were calculated for the detection of salinity as given in Equations (5)–(8).

When making predictions in regression, R${}^{2}$ measures the proportion of variance in the output or dependent variable that can be explained by the input or independent variables. It ranges from 0 to 1. A value of 1 indicates a perfect fit, implying that the model precisely captures the variance in the dataset. Conversely, a value of 0 suggests that the model does not explain any variance in the data and is not a good fit for the dataset [76].

(5) $$R^{2}=1-\frac{\sum_{i=1}^{n}{\left(y_{i}-{\hat{y}}_{i}\right)}^{2}}{\sum_{i=1}^{n}{\left(y_{i}-{\bar{y}}_{i}\right)}^{2}}$$

Here, $y_{i}$ is the ith observed salinity, ${\hat{y}}_{i}$ is the ith predicted salinity, ${\bar{y}}_{i}$ is the average salinity of all the soil samples, and *n* is the total number of samples.

MAE is a metric employed to evaluate the average absolute difference between the actual values and the predicted values within a dataset. For a better model and good accuracy, its value should be close to 0 [76].

(6) $$MAE=\frac{\sum_{i=1}^{n}\left|y_{i}-x_{i}\right|}{n}$$

MSE shows the mean or average of the squared differences between the actual and predicted values in a dataset. RMSE is simply the square root of MSE [76].

(7) $$MSE=\sum_{i=1}^{n}\frac{{\left({\hat{y}}_{i}-y_{i}\right)}^{2}}{n}$$

RMSE is used to measure the error of a model in predicting the results. Predictions are good when its value is close to zero [76].

(8) $$RMSE=\sqrt{\sum_{i=1}^{n}\frac{{\left({\hat{y}}_{i}-y_{i}\right)}^{2}}{n}}$$

Here, n is the number of observations or samples. $y_{1},y_{2}$,…, $y_{n}$ are observed values. ${\hat{y}}_{1},{\hat{y}}_{2}$,…, ${\hat{y}}_{n}$ are predicted values.

## 3. Results

### 3.1. Satellite Data Sensitivity to Soil Type

#### MODIS Terra Data Sensitivity Analysis

**Figure 3:**
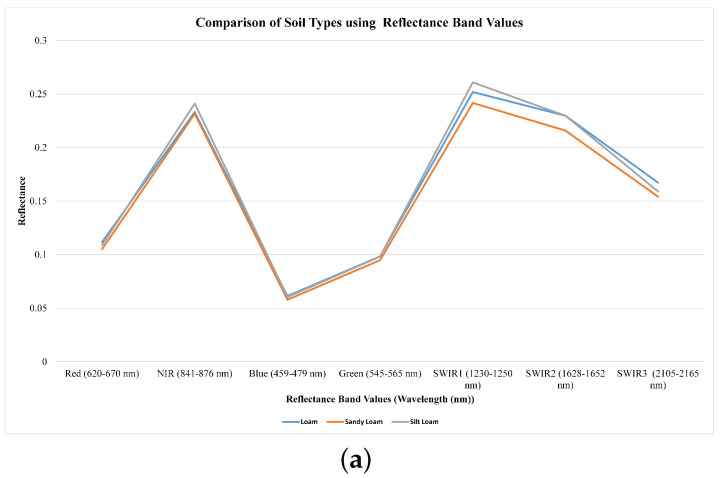

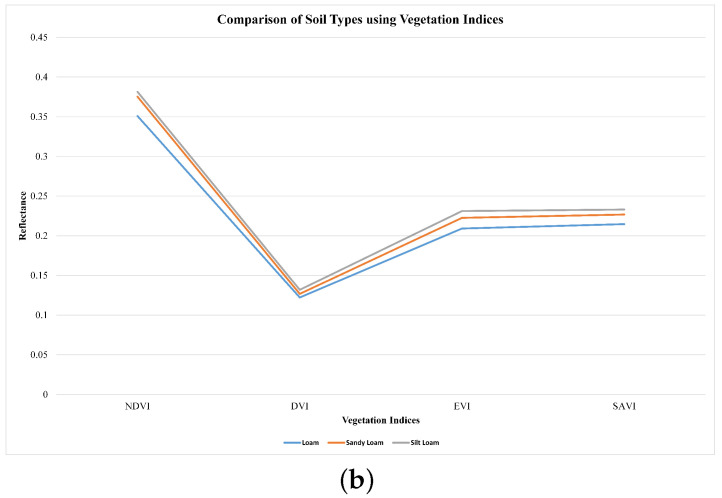
Comparison of Soil Types (**a**) using mean of reflectance band values (**b**) using vegetation indices.

An investigation was carried out to assess how MODIS Terra images across seven distinct bands (sur_refl_b01-sur_refl_b07) and various vegetation indices influenced the classification of soil types. To predict soil salinity levels, eight salinity indices were incorporated alongside these spectral bands. The results revealed that the reflectance values in bands 1, 3, and 4 remained relatively consistent for Loam, Sandy Loam, and Silt Loam soil types. Conversely, bands 2, 5, 6, and 7 exhibited varying degrees of sensitivity in distinguishing between different soil types, as depicted in (Figure 3a). Efficient discrimination of soil types was achieved through the analysis of reflectance values in band 2, band 5, and band 7. Specifically, Silt Loam displayed notably higher reflectance values in band 2 and band 7 when compared to Loam and Sandy Loam soil types. The reflectance value in band 5 also played a crucial role in distinguishing between Loam, Sandy Loam, and Silt Loam soil types. Likewise, other studies have also explored the efficacy of NIR (band 2) and SWIR bands (bands 5–7) in assessing soil properties [19,20,21]. The variations in reflectance among the visible, NIR, and SWIR bands in MODIS, used for classifying soil types, mainly arise from the diverse interplay between soil characteristics, surface conditions, and electromagnetic radiation at different wavelengths. Furthermore, we evaluated the sensitivity of soil types using various vegetation indices, such as NDVI, DVI, EVI, and SAVI. The reflectance values of the DVI vegetation index remained consistent across Loam, Sandy Loam, and Silt Loam soil types. In contrast, when employing NDVI, EVI, and SAVI, Silt Loam consistently exhibited higher reflectance values in comparison to Loam and Sandy Loam, as shown in Figure 3b.

### 3.2. Classification Scheme

Based on the spectral band values, we computed vegetation indices to create our dataset. This dataset includes eleven attributes and a class label called “soil type” for the purpose of soil type classification. The vegetation indices generated for the soil types encompass NDVI, DVI, EVI, and SAVI. Through sensitivity analysis, we determined that all these indicators exhibited sensitivity to the different soil type classes as shown in Figure 3. Therefore, all spectral bands were included in the classification process. In the current study, the classification dataset consists of five classes: Silt Loam, Loam, Sandy Loam, Sandy Clay Loam, and Clay Loam. Here are the details regarding the soil tuples within the dataset, as given in Table 2. The dominant classes within the dataset were Loam, Sandy Loam, and Silt Loam, while Clay Loam and Sandy Clay Loam tuples were relatively scarce. Consequently, the tuples belonging to Clay Loam and Sandy Clay Loam were disregarded. Subsequently, this labeled dataset, comprising a total of 184 tuples, was employed to train a supervised machine learning model. This model was designed to categorize specific land areas into different land types based on their soil characteristics.

### 3.3. Validation

**Figure 4:**
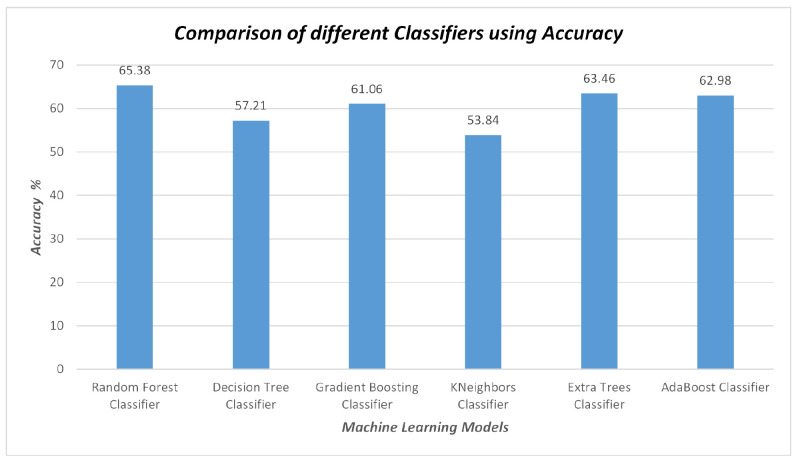
Comparison of the Algorithm’s Accuracies.

**Figure 5:**
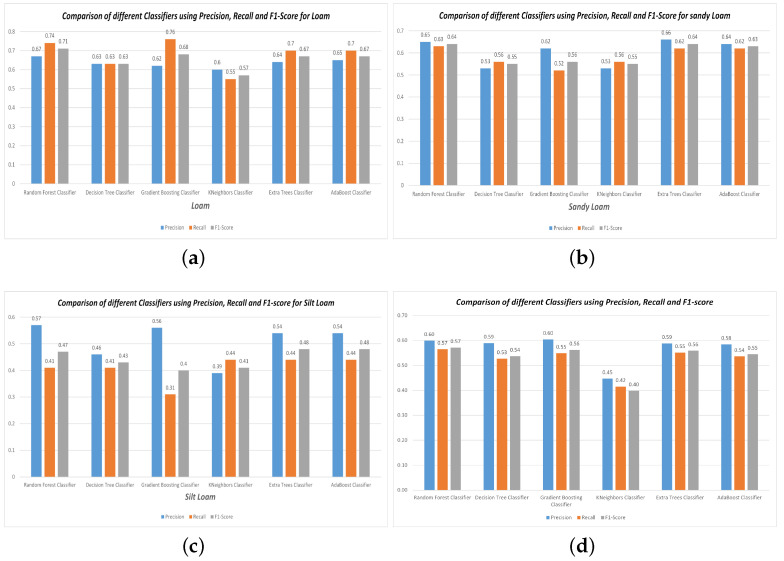
Comparison of Classifiers precision, recall and f1-score: (**a**) Loam (**b**) Sandy Loam (**c**) Silt Loam (**d**) Overall.

Band values and vegetation indices were used as input for these classifiers, with the observed soil type as the target value. The dataset was split into testing and training sets at a ratio of 70% for training and 30% for testing. To assess the performance of the classification models, various evaluation metrics were employed, including accuracy, precision, recall, and f1-score. The accuracy values of the classifiers, derived from the confusion matrices, are provided in Figure 4. The RF model demonstrated strong performance with an accuracy of 65.38% when identifying soil types. The results derived from MODIS Terra data indicate that the RF model stands out as a more robust classifier compared to GB, AB, DT, KNN, and ET in all the experiments conducted during the study as shown in Figure 4 and Figure 5.

The precision, recall, and f1-score comparisons for these models regarding Loam, Sandy Loam, and Silt Loam are shown in Figure 5.

Considering the accuracy, precision, recall, and f1-score values, it is apparent that both the RF and GB classifiers outperformed all the other classifiers, achieving accuracy of 65.38% and 61.06%, respectively.

### 3.4. Regression for Soil Salinity

**Figure 6:**
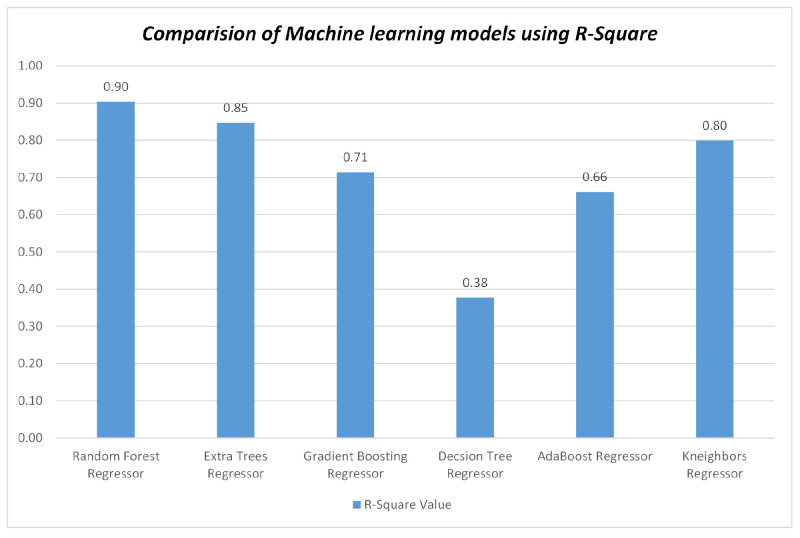
Comparison of the algorithm’s using R${}^{2}$ values.

To predict soil salinity, we utilized the reflectance values from seven bands and included eight salinity indices as input parameters. This analysis was conducted using a dataset containing 184 soil samples, which was partitioned into a calibration set (70%) and a testing set (30%). The performance of the regression models was assessed using various evaluation metrics, including R${}^{2}$, MAE, MSE, and RMSE. In the present study, the RF model demonstrated the strongest correlation with the highest R${}^{2}$ value of 0.90 when compared to other models, including GB, AB, DT, KNN, and ET, as shown in Figure 6. This model showcases superior efficiency due to its notably low MAE, MSE, and RMSE values. Consequently, the RF model stands out as the optimal regressor, boasting impressive performance with MAE at 0.56, MSE at 0.98, and RMSE at 0.97.

**Figure 7:**
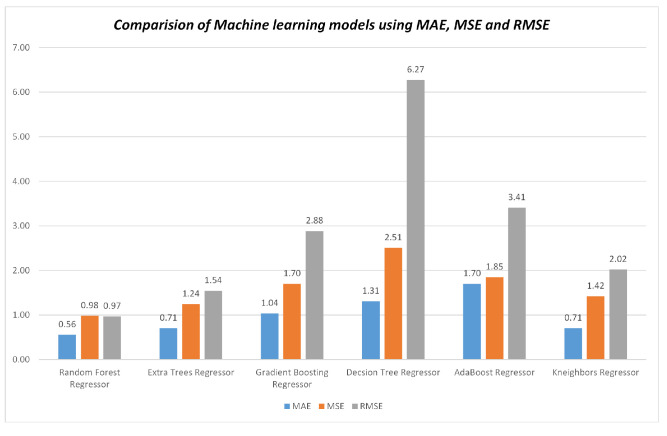
Comparison of the algorithms using MAE, MSE, and RMSE .

**Figure 8:**
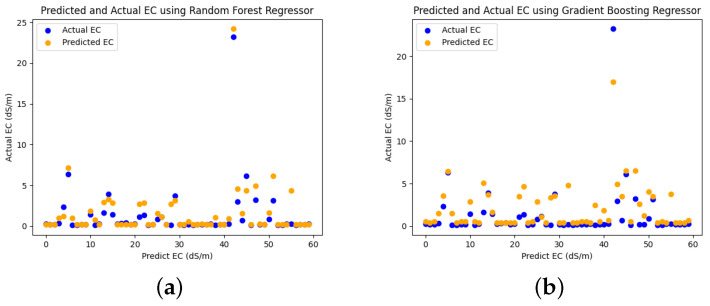

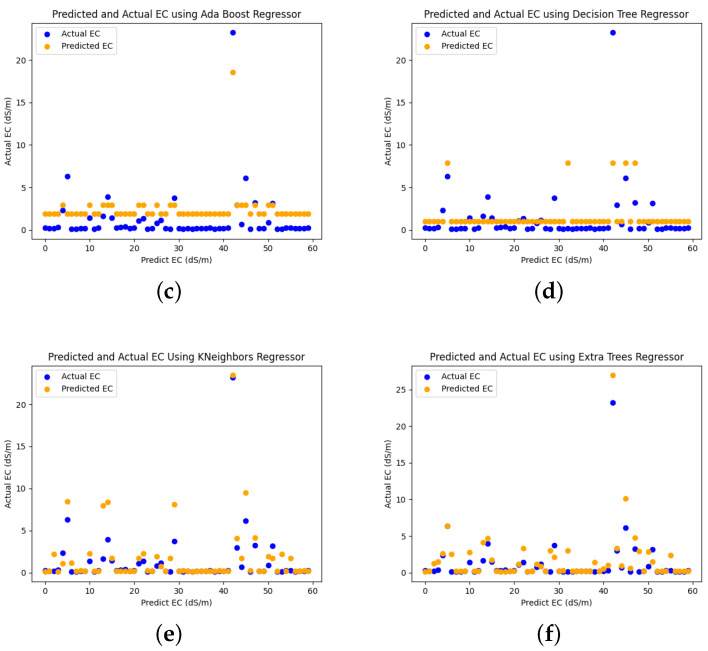
Scatter plots of predicted and actual EC values using: (**a**) RF (**b**) GB (**c**) AB (**d**) DT (**e**) KNN (**f**) ET.

The strong alignment between the actual and predicted soil salinity, often represented as Electric Conductivity (EC) values, suggests a robust model fit. Specifically, when compared to GB, AB, DT, KNN, and ET, the RF model demonstrated a notably closer alignment between its actual and predicted EC values, as illustrated in Figure 8.

**Figure 9:**
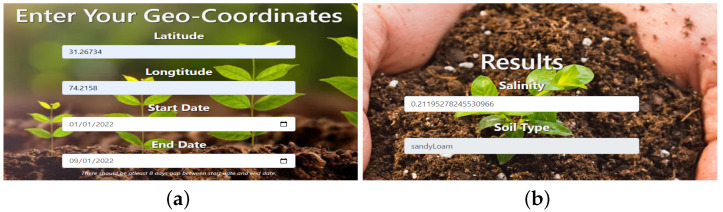

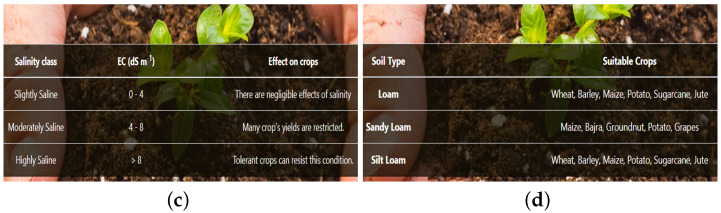
Web portal: (**a**) Enter geocoordinate and select dates (**b**) Soil types and salinity (**c**) salinity ranges (**d**) suitable crops based on soil type.

Based on the study’s findings, a user-friendly web portal was created. This portal enables users to estimate soil types and salinity effortlessly by entering geographic coordinates and selecting preferred dates. Once the user clicks the submit button, the system accesses MODIS Terra data images from the USGS website. Subsequently, it calculates seven spectral bands, vegetation indices, and salinity indices to determine soil types and forecast soil salinity levels. Additionally, this web portal offers insights into the effects of soil salinity on crops and suggests appropriate crop choices based on the identified soil types. The interface of this portal is illustrated in Figure 9. This innovative tool not only provides a quick and cost-efficient method for identifying soil type and salinity but also functions as a valuable resource for farmers and agricultural officers. It empowers them to access real-time information about their land conditions.

## 4. Discussion

To assess the effectiveness of different ML algorithms for soil type classification and salinity detection, we compared the performance of RF, GB, AB, DT, KNN, and ET using MODIS Terra data. The RF model demonstrated superior performance with an accuracy of 65.38% while mapping the soil types, outperforming the other models, as shown in Figure 4. Moreover, the RF model exhibited higher precision, recall, and f1-score compared to GB, AB, DT, KNN, and ET, as depicted in Figure 5. Similarly, the effectiveness of RF in soil type classification was evident in the preliminary study [41]. Additionally, the RF model proved to be the most suitable for soil salinity mapping, achieving R${}^{2}$ of 0.90, MAE of 0.56, MSE of 0.98, and RMSE of 0.97, as shown in Figure 6 and Figure 7. The RFR model demonstrated superior performance compared to the GB, AB, DT, KNN, and ET, primarily because of its utilization of random sampling [77], its effective fitting on smaller datasets [78], and the resulting improvement in decision-making accuracy [55]. Indeed, the preliminary studies provided clear evidence of the effectiveness of RF in predicting soil salinity [38,79].

In our second research objective, where we assessed the accuracy of soil type and salinity mapping using reflectance values and vegetation indices from MODIS Terra data, we investigated how these parameters influenced various soil type categories. In the context of soil salinity mapping, we incorporated eight salinity indices in combination with seven spectral signatures. Our findings indicated that the reflectance values of band 1, band 3, and band 4 displayed relatively consistent patterns across Loam, Sandy Loam, and Silt Loam soil texture classes. Conversely, the reflectance values of band 2, band 5, band 6, and band 7 exhibited sensitivity in distinguishing between different soil types, as depicted in Figure 3a. Specifically, the reflectance values in band 2, band 5, and band 7 were particularly effective in distinguishing between various soil types. These spectral signatures of reflectance values played a pivotal role in achieving accurate soil type mapping, as demonstrated in Figure 3a. Remarkably, the Silt Loam soil type exhibited higher reflectance values in band 2 and band 7 in comparison to Loam and Sandy Loam soil types. Moreover, the reflectance value in band 5 played a crucial role in distinguishing between Loam, Sandy Loam, and Silt Loam soil types. Additionally, we conducted an analysis to assess the sensitivity of soil types using vegetation indices such as NDVI, DVI, EVI, and SAVI. The reflectance values of the DVI vegetation index remained relatively consistent across Loam, Sandy Loam, and Silt Loam soil types. However, when considering NDVI, EVI, and SAVI, Silt Loam exhibited higher reflectance values than Loam and Sandy Loam, as seen in Figure 3b. Our results revealed that the NIR and SWIR bands exhibited higher sensitivity in distinguishing between soil type classes, as illustrated in Figure 5. Similarly, other studies have investigated the effectiveness of NIR (band 2) and SWIR bands (bands 5–7) for evaluating soil characteristics [19,20,21]. Likewise, soil salinity was effectively mapped with R${}^{2}$ of 0.90, MAE of 0.56, MSE of 0.98, and RMSE of 0.97 using seven reflectance band values and eight salinity indices with the RF model, as shown in Figure 6. The scatter plots in Figure 8 also demonstrate the close alignment between the EC predicted and actual values when using the RF model.

This research study significantly advances the understanding of soil types and salinity across various regions in Pakistan by leveraging remote sensing data and machine learning techniques. Our findings demonstrate promising outcomes, with a 65.38% accuracy rate for soil type mapping and an R${}^{2}$ value of 0.90 for soil salinity prediction using the RF model. Moreover, we have developed a user-friendly web portal, as illustrated in Figure 9, which enables effortless determination of soil type and salinity for barren land simply by entering geographical coordinates. This portal holds the potential to offer valuable insights to farmers, assisting them in making informed decisions regarding crop selection and implementing strategies to mitigate soil erosion.

## 5. Conclusions

Identifying soil types and assessing soil salinity are essential for optimizing crop cultivation in particular geographic areas. Pakistan, an agricultural-oriented nation, still depends on traditional methods for classifying soil types and measuring soil salinity. This current investigation aims to create a model using MODIS Terra data to categorize soil types and predict the level of soil salinity. To train the machine learning models for soil type classification and soil salinity prediction, a total of 184 samples were gathered from barren land across three distinct study regions. It was observed that the NIR and SWIR bands, in conjunction with vegetation indices like NDVI, EVI, and SAVI, exhibited greater sensitivity in distinguishing between Loam, Sandy Loam, and Silt Loam soil types. Six widely recognized machine learning techniques (RF, GB, AB, DT, KNN, and ET) were utilized to classify soil types and forecast soil salinity. The outcomes indicated that the RF model performed the most effectively, achieving an accuracy rate of 65.38% for soil type classification (with precision at 0.60, recall at 0.57, and an f1-score of 0.57) and an R${}^{2}$ value of 0.90 for soil salinity prediction (with MAE at 0.56, MSE at 0.98, and RMSE at 0.97), surpassing the performance of other models (RF, GB, AB, DT, KNN, and ET). The combination of remote sensing technology and machine learning models demonstrates its efficiency in evaluating soil types and salinity at a local scale. To streamline the process, a user-friendly web portal was developed based on this research, enabling users to determine soil types and salinity by simply entering geographical coordinates. This information can prove invaluable for farmers and agricultural management in selecting suitable crop varieties and minimizing potential financial losses stemming from climate change. The approach outlined in this study is both rapid and cost-effective for identifying soil types and assessing salinity in specific regions. While this research relies on freely available MODIS Terra data, future enhancements could involve expanding the dataset with more soil samples, study regions, and the application of deep learning models to further enhance accuracy.

## Figures and Tables

**Table 1:** Spectral Indices for Current Study.

Spectral Indices	Expression	References
	**Vegetation Indices**	
NDVI	$\frac{\left(NIR\;-\;Red\right)}{\left(NIR\;+\;Red\right)}$	Rouse et al. [44]
DVI	NIR − Red	Basso et al. [45]
EVI	$2.5\;\times \;\frac{NIR\;-\;Red}{NIR\;+\;6\;\times \;Red\;-\;7.5\;\times \;Blue\;+\;1)}$	Liu and Huete [46]
SAVI	$\frac{\left(NIR\;-\;Red\right)}{\left(NIR\;+\;Red\;+\;L\right)}\times \left(L\;+\;1\right)$	Huete [47]
	**Salinity Indices**	
NDSI	$\frac{\left(Red\;-\;NIR\right)}{\left(Red\;+\;NIR\right)}$	Khan et al. [48]
VSSI	2 × Green − 5 × (Red + NIR)	Dehni and Lounis [49]
SI	$\frac{\left(Red\;\times \;Green\right)}{Blue}$	Allbed et al. [50]
SI1	$\sqrt{Green\;\times \;Red}$	Abd El Kader Douaoui and Walter [51]
SI2	$\sqrt{Red\;\times \;NIR}$	Dehni and Lounis [49]
SI3	$\sqrt{{\left(Green\right)}^{2}\;+\;{\left(Red\right)}^{2}\;+\;{\left(NIR\right)}^{2}}$	Abd El Kader Douaoui and Walter [51]
SI4	$\sqrt{{\left(Green\right)}^{2}\;+\;{\left(Red\right)}^{2}}$	Yahiaoui et al. [52]
SI5	$\frac{Blue}{Red}$	Abbas and Khan [53]

**Table 2:** Details of Classification Dataset.

Sr. No	Soil Type	Instances
1	Silt Loam	55
2	Loam	70
3	Sandy Loam	61
4	Sandy Clay Loam	4
5	Clay Loam	7
Total		195

## Data Availability

Upon reasonable request, the underlying data for this article will be made available by the corresponding author.
